# Characteristic profiles of molecular types, antibiotic resistance, antibiotic resistance genes, and virulence genes of *Staphylococcus aureus* isolates from caprine mastitis in China

**DOI:** 10.3389/fcimb.2025.1533844

**Published:** 2025-02-18

**Authors:** Hongfei Shi, Long Wang, Guoguang Li, Dandan Li, Hongyue Zhai, Shidong Ji, Yun Hu, Tingting Lv, Lunguang Yao

**Affiliations:** ^1^ Henan Provincial Engineering and Technology Center of Animal Disease Diagnosis and Integrated Control, Henan Provincial Engineering Laboratory of Insects Bio-reactor, Nanyang Normal University, Nanyang, China; ^2^ Henan Province Engineering Technology Research Center of Animal Disease Control and Prevention, Nanyang Vocational College of Agriculture, Nanyang, China

**Keywords:** *Staphylococcus aureus*, mastitis, goat, antimicrobial resistance, virulence gene

## Abstract

*Staphylococcus aureus* is a significant pathogen in dairy animals, particularly when it infects the mammary gland; however, its prevalence among dairy goats in China remains poorly understood. This study aimed to investigate the distribution and characteristics of *S. aureus* isolates in dairy goats across China. A total of 515 milk samples were collected from goats diagnosed with mastitis in 14 provinces. These samples underwent bacterial isolation and identification, capsular polysaccharides typing, spa typing, antimicrobial susceptibility testing, and assessment of antimicrobial resistance and virulence gene. The findings revealed the isolation of 61 *S. aureus* strains. The highest prevalence rate was recorded in 2018, at 20.4% (11 out of 54 samples), while the lowest prevalence rate was noted in 2023, at 5.2% (3 out of 58 samples). Among the five regions studied, southern China exhibited the highest prevalence rate of 17.5% (10 out of 57 samples), whereas northeastern China showed the lowest rate at 8.2% (8 out of 97 samples). Capsular polysaccharide type 5 emerged as the most prevalent, accounting for 52.5%, and spa type t521 was identified most frequently, at 19.7%. Notably, 52 isolates (85.2%) demonstrated multidrug resistance, displaying resistance to three or more antibiotics. The resistance rates of *S. aureus* isolates were significantly high to penicillin (95.1%), followed by enrofloxacin (82.0%), kanamycin (78.7%), and levofloxacin (77.0%). Trimethoprim-sulfamethoxazole exhibited the lowest resistance rate at 11.5%. Resistance rates varied across the five different regions. Additionally, eight genes associated with resistance to six classes of antimicrobials were detected, with the *blaZ* gene (93.4%) being the most prevalent at 93.4%. Furthermore, nine virulence-associated genes were identified, with clfA being the most common virulence gene, present in all isolates. In conclusion, most *S. aureus* isolates were multiresistant with diverse resistance patterns. Those diverse antimicrobial resistance profiles associated with corresponding resistance genes (*p* < 0.05) were reported for the first time in *S. aureus* from caprine mastitis. Sulfonamides could be prioritized preferentially for the treatment of *S. aureus* mastitis.

## 1 Introduction

Mastitis refers to the inflammation of the mammary glands, which can be attributed to various pathogenic factors. Clinical mastitis may result in a reduction in milk production, diminished milk quality, udder gangrene, loss of lactation capability, prolonged estrus, and decreased overall production performance, ultimately leading to significant economic losses (Olechnowicz and Jaśkowski, 2014). In China, the prevalence of mastitis in dairy goats has been reported to be 45.82% (Zhao et al., 2015). Bacterial infection is the primary etiological factor associated with mastitis, with *Staphylococcus aureus* being one of the most significant pathogens. *S. aureus* is a species of Gram-positive, grape-forming coccus. To date, the presence of *S. aureus* in dairy cattle suffering from mastitis has been documented in numerous reports within China (Gao et al., 2017; Cheng et al., 2019; Song et al., 2020; Song et al., 2024). A meta-analysis, based on 4,215 bovine mastitis samples in 33 studies, revealed a pooled prevalence of *S. aureus* at 36.23% (Wang et al., 2022). In contrast, the epidemiology of *S. aureus* in dairy goats with mastitis has been reported in only a limited number of studies (Zhao et al., 2015; Liu et al., 2023). It is noteworthy that China hosts over 1,290,000 dairy goats (Luo et al., 2019). In 2015, the prevalence rates of *S. aureus* in samples collected from dairy goats were reported to be 12.82%, 22.58%, and 14.85%, respectively, in three provinces (Shandong, Yunnan, and Shaanxi) in China (Zhao et al., 2015). Recently, in Shaanxi province, the prevalence of *S. aureus* was observed in 58.33% of raw milk samples (28/48) collected from dairy goats with clinical mastitis (Liu et al., 2023). However, the characteristics of *S. aureus* strains have not been documented in the aforementioned reports.

To date, the capsular polysaccharides (CP) of *S. aureus* have been classified into 11 types, of which only CP5 and CP8 types (encoded by the genes *cap5* and *cap8*, respectively) are present in more than 80% of clinical strains (Rozemeijer et al., 2015). In addition, spa typing, another widely utilized typing method, relies on the tandem repeats and sequence variations in the protein A gene region X (Moodley et al., 2006). Spa typing employs a standardized international nomenclature based on single-base sequence analysis, which is convenient for probing molecular correlations between *S. aureus* from different hosts, environments, sources, and countries (Rodriguez et al., 2015). To our knowledge, no studies have been conducted on the prevalent types of *S. aureus* strains from dairy goats in China; thus, molecular epidemiology data are not available.

Currently, antimicrobial therapy is the primary method for preventing and treating *S. aureus* infections in dairy herds. Unfortunately, the extensive use of antimicrobial agents has led to the emergence of resistance to various antibiotics through phenotype changes, rendering these antibiotics increasingly ineffective (Bujňáková and Karahutová, 2024; Hutu et al., 2024; Shahzad et al., 2024; Zhu et al., 2024). In China, national antimicrobial resistance monitoring and surveillance programming in animals have been carried out for many years. However, information of the antibiotic resistance of *S. aureus* from dairy goats remains scarce. The resistance of *S. aureus* is closely related to drug resistance genes; for example, *blaZ* and *mecA* are considered the main genes responsible for β-lactam resistance, and *tetK* and *tetM* account for tetracycline resistance (Al-Ashmawy et al., 2016; Merz et al., 2016; Akkou et al., 2018). Moreover, since resistance genes may transfer from *S. aureus* to the indigenous microbiota in the human gut (Lee John, 2003), this undoubtedly increases the huge public health risk.

The pathogenicity of *S. aureus* multiplies, influenced by a variety of virulence factors, which included both structural components of the bacteria and secreted factors (Wang et al., 2018). For instance, bacterial adhesion to the epithelial cells of the teat canal is mediated by clumping factors A and B (*clfA* and *clfB*) as well as fibronectin-binding protein A (*fnbA*) (Bochniarz et al., 2016). Furthermore, the secreted virulence factors encompass over 20 different enterotoxins, which are traditionally categorized into classical genes from *sea* to *sem*, along with other significant factors, such as the toxic shock syndrome toxin (TSST-1) and staphylococcal exfoliative toxin (ET). These factors play a crucial role in the pathogenesis of mastitis caused by *S. aureus* (Costa et al., 2018; Mohseni et al., 2018).

The aim of the present study is to investigate the distribution of *S. aureus* isolates in cases of mastitis in dairy goat mastitis and to identify the presence of resistance and virulence genes in these isolates collected from 2015 to 2024. The findings of this study will contribute to the scientific foundation for the development of prevention and control strategies in the production of dairy goats in China.

## 2 Materials and methods

### 2.1 Sample collection, bacterial isolation, and identification

From 2015 to 2024, a total of 515 batches of raw milk were collected from dairy goats diagnosed with clinical mastitis across 32 farms located in 14 provinces in China (**Table 1**). The dairy goats involved in this study were raised in backyard farms utilizing a free-range system, with herd sizes ranging from 20 to 60 animals. Milking was predominantly conducted manually twice daily. It is noteworthy that teat disinfection practices on these farms were irregular; specific details regarding the cleaning agents used, their concentrations, mode of application, and frequency of cleaning are provided in **Supplementary Table 1**. All milk samples were collected under aseptic conditions, transferred to sterile tubes, and stored in an ice box at 4°C for transportation to the laboratory for bacterial analysis.

**Table 1:** Goat milk samples with clinical mastitis collected from backyard dairy goat farms in five regions of China from 2015 to 2024.

Region	Province	Farm	Breed	Samples, *n*	Samples with confirmed *Streptococcus aureus*	
					By herd, *n* (%)	By region, %
Northeast China	Heilongjiang	1	Saanen	14	1 (7.1)	8/97 (8.2)
		2	Saanen	18	1 (11.1)	
	Liaoning	3	Saanen	13	1 (15.4)	
		4	Saanen	21	1 (7.7)	
	Jilin	5	Saanen	12	2 (16.7)	
	Inner Mongolia	6	Guanzhong	19	2 (10.5)	
Northwest China	Shanxi	7	Xinong Saanen	22	3 (13.6)	11/100 (11.0)
		8	Guanzhong	18	2 (10.5)	
		9	Xinong Saanen	25	2 (8.0)	
		10	Xinong Saanen	19	3 (15.8)	
	Gansu	11	Xinong Saanen	16	1 (6.3)	
Central China	Henan	12	Xinong Saanen	17	3 (17.6)	26/217 (12.0)
		13	Guanzhong	22	2 (9.1)	
		14	Xinong Saanen	14	2 (14.3)	
		15	Xinong Saanen	10	1 (10.0)	
		16	Guanzhong	13	3 (23.1)	
		17	Xinong Saanen	23	3 (13.0)	
		18	Guanzhong	15	1 (6.7)	
		19	Xinong Saanen	11	2 (18.2)	
		20	Xinong Saanen	17	2 (11.8)	
		21	Guanzhong	16	1 (6.3)	
	Hubei	22	Xinong Saanen	15	2 (13.3)	
		23	Guanzhong	11	2 (18.2)	
		24	Guanzhong	20	1 (5.0)	
		25	Xinong Saanen	13	1 (7.7)	
Eastern China	Shandong	26	Laoshan	14	3 (21.4)	6/44 (13.6)
		27	Laoshan	18	2 (11.1)	
	Anhui	28	Saanen	12	1 (8.3)	
Southern China	Guizhou	29	Saanen	13	2 (15.4)	10/57 (17.5)
	Yunnan	30	Guishan	11	3 (27.3)	
	Hunan	31	Saanen	17	3 (17.6)	
	Jiangsu	32	Saanen	16	2 (12.5)	
Total				515		61/515 (11.8)

Milk samples were inoculated onto blood agar plates supplemented with 5% defibrinated sheep blood and incubated at 37°C for 24 to 48 h. Subsequently, typical colonies were identified through Gram staining and catalase testing. Suspected *S. aureus* isolates were confirmed by detecting the thermonuclease (*nuc*) gene, which is specific to this bacterium (Brakstad et al., 1992). In summary, DNA was extracted using a Bacteria Genomic DNA kit (TransGen Biotech, China) in accordance with the manufacturer’s instructions. The extracted DNA was dissolved in 100 µL of ultrapure water and stored at −70°C for subsequent PCR analysis. In addition, PCR products from five *nuc* positive strains were randomly selected for sequencing to ensure further verification. Following identification, one single *nuc* gene positive isolate from each sample was selected and stored for subsequent antimicrobial susceptibility testing. *S. aureus* ATCC25923 was used as a quality control strain. The prevalence rate was calculated as the following formula: prevalence rate = (number of existing cases/total sample number) × 100%.

### 2.2 Typing of *S. aureus*

Both capsular polysaccharide typing and spa typing were used to type all stored *S. aureus* strains. According to the previous study (Verdier et al., 2007), two primer sets were selected for capsular polysaccharide typing, namely, *Cap5* k1 (5′-GTCAAAGATTATGTGATGCTACTGAG-3′) and *Cap5* k2 (5′-ACTTCGAATATAAACTTGAATCAATGTTATACAG-3′) located in *cap5k*, specific for capsular type 5, and *cap8 k1* (5′-GCCTTATGTTAGGTGATAAACC-3′) and *cap8* k2 (5′-GGAAAAACACTATCATAGCAGG-3′) located in *cap8I*, specific for capsular type 8.

Molecular typing of spa types was also carried out via PCR using the primer set including spa-1113f (5′-TAAAGA CGATCC TTC GGTGAGC-3′) and spa-1514r (5′-CAG CAGTAGTGCCGTTTGCTT-3′). The results were submitted to the website for further detection. Then, these results were interpreted according to the guidelines described at the Ridom SpaServer database (www.spaserver.ridom.de).

### 2.3 Antimicrobial susceptibility testing

The antibiotic resistance patterns of each nuc-positive *S. aureus* isolate from the samples (selected and stored in Section 2.1) were determined by disk diffusion methods according to Clinical and Laboratory Standards Institute (CLSI) guidelines (CLSI, 2018). The inhibition zones were measured and recorded, with breakpoints for various antibiotics determined based on CLSI guidelines (CLSI, 2018). In instances where specific antibiotic testing and interpretation guidelines were unavailable, the instructions for antibiotic-sensitive papers (Hangzhou Microbial Reagent Company, China) were used as a reference. Resistance to 18 antimicrobials belonging to eight antimicrobial categories was investigated in the drug susceptibility test. Each experiment was repeated at least three times to ensure reproducibility, and *S. aureus* ATCC25923 served as a quality control strain. Moreover, isolates exhibiting resistance to three or more antimicrobial categories were classified as multidrug resistant (Moodley et al., 2006).

### 2.4 Detection of resistance genes

Bacterial genomic DNA was extracted as described in the section on bacterial identification. The detection of antibiotic resistance genes and virulence genes was performed using conventional PCR. In alignment with the categories of antimicrobials utilized in this work and the most commonly identified resistance genes in *S. aureus* as reported in previous research conducted in China (Zhang et al., 2022; Ning et al., 2023; Zhu et al., 2024), the following resistance genes were detected: β-lactam resistance genes *mecA*, *blaZ*, *femA*, and *femB* (Kobayashi et al., 1994; Vannuffel et al., 1998; Choi et al., 2003; Olsen et al., 2006); tetracycline resistance genes *tetK* and *tetM* (Strommenger et al., 2003); macrolide resistance genes *ermA*, *ermB*, and *ermC* (Sutcliffe et al., 1996); phenicols resistance gene *fexA* (Argudín et al., 2011); sulfonamide resistance gene *dfrD* (Argudín et al., 2011); aminoglycoside resistance genes *aacA-aphD* (Strommenger et al., 2003); the lincosamide resistance gene *lnu(B)* (formerly known as *linB*) (Bozdogan et al., 1999); and quinolone resistance genes *gyrA* and *parC* (Schmitt-Van-de-Leemput and Zadoks, 2007). Detailed information regarding all resistance gene primers, including product size and annealing temperature, is presented in **Table 2**.

**Table 2:** Primers of resistance genes.

Gene name	Sequence (5′-3′)	Product size (bp)	Annealing temperature (°C)	Reference
*blaZ*	F: TAAGAGATTTGCCTATGCTT	377	48	(Olsen et al., 2006)
	R: TTAAAGTCTTACCGAAAGCAG			
*mecA*	F: GTGAAGATATACCAAGTGATT	147	57	(Choi et al., 2003)
	R: GTGAAGATATACCAAGTGATT			
*femA*	F: CTTACTTACTGGCTGTACCTG	686	58	(Vannuffel et al., 1998)
	R: ATGTCGCTTGTTATGTGC			
*femB*	F: TTACAGAGTTAACTGTTACC	651	57	(Kobayashi et al., 1994)
	R: ATACAAATCCAGCACGCTCT			
*dfrD*	F: CCCTGCTATTAAAGCACC	606	52	(Argudín et al., 2011)
	R: CATGACCAGATAACTC			
*dfrK*	F: CAAGAGATAAGGGGTTCAGC	229	51	(Argudín et al., 2011)
	R: ACAGATACTTCGTTCCACTC			
*dfrG*	F: TGCTGCGATGGATAAGAA	405	54	(Argudín et al., 2011)
	R: TGGGCAAATACCTCATTCC			
*tetM*	F: AGTGGAGCGATTACAGAA	158	48	(Strommenger et al., 2003)
	R: CATATGTCCTGGCGTGTCTA			
*tetK*	F: GTAGCGACAATAGGTAATAGT	360	48	(Strommenger et al., 2003)
	R: GTAGTGACAATAAACCTCCTA			
*ermA*	F: TCTAAAAAGCATGTAAAAGAA	645	45	(Sutcliffe et al., 1996)
	R: CTTCGATAGTTTATTAATATTAGT			
*ermB*	F: GAAAAGGTACTCAACCAAATA	639	45	(Sutcliffe et al., 1996)
	R: AGTAACGGTACTTAAATTGTTTAC			
*ermC*	F: TCAAAACATAATATAGATAAA	642	45	(Sutcliffe et al., 1996)
	R: GCTAATATTGTTTAAATCGTCAAT			
*fexA*	F: GTACTTGTAGGTGCAATTACGGCTGA	1272	57	(Argudín et al., 2011)
	R: CGCATCTGAGTAGGACATAGCGTC			
*aacA-aphD*	F: GAAGTACGCAGAAGAGA	491	45	(Strommenger et al., 2003)
	R: ACATGGCAAGCTCTAGGA			
*lnu(B)*	F: CCTACCTATTGTTTGTGGAA	925	54	(Bozdogan et al., 1999)
	R:ATAACGTTACTCTCCTATTC			
*gryA*	F: AATGAACAAGGTATGACACC	223	50.5	(Schmitt-Van-de-Leemput and Zadoks, 2007)
	R: TACGCGCTTCAGTATAACGC			
*parC*	F:A CTTGAAGATGTTTTAGGTGAT	559	56	(Schmitt-Van-de-Leemput and Zadoks, 2007)
	R: CTTGAAGATGTTTTAGGTGAT			

An EasyTaq^®^ PCR SuperMix kit (TransGen, Beijing, China) was used for PCR reactions. A total reaction mixture was 20 μL, comprising 10 μL of 2× EasyTaq^®^ PCR SuperMix, 0.4 μM of each primer, and 20 ng of template DNA. Then, these mixtures were denatured at 94°C for 5 min, followed by 30 cycles of 30 s at 94°C, 30 s at an appropriate annealing temperature determined by the specific resistance and virulence gene primers, 30 s at 72°C, and a final extension at 72°C for 10 min. Meanwhile, samples with goat DNA or without genomic DNA were included as controls. The amplified products were subjected to electrophoresis through a 2% agarose gel (Solarbio, China) at 120 V for 60 min.

### 2.5 Detection of virulence genes

Several genes associated with virulence were screened via PCR, including the staphylococcal adhesion factors *fnbA*, *clfA*, *clfB*, and *ebpS*; enterotoxin (*sea*, *seb*, *sec*, *sed*, *see*, *seg*, *seh*, *sei*, *sej*, *sen*, *seo*, and *sem*); poisoning syndrome toxin (*tst*); and shedding toxin (*eta*, *etb*) (Jarraud et al., 2002; Peacock et al., 2002). The primers for all virulence genes, along with their respective product sizes and annealing temperatures, are presented in **Table 3**. The PCR protocols employed were consistent with the previously described protocols in Section 2.4.

**Table 3:** Primers of virulence genes.

Gene name	Sequence (5′-3′)	Product size (bp)	Annealing temperature (°C)	Reference
*fnbA*	F: CACAACCAGCAAATATAG	1,362	50	(Jarraud et al., 2002)
	R: CTGTGTGGTAATCAATGTC			
*clfA*	F: GTAGGTACGTTAATCGGTT	1,584	45	(Jarraud et al., 2002)
	R: CTCATCAGGTTGTTCAGG			
*clfB*	F: TGCAAGATCAAACTGTTCCT	596	45	(Jarraud et al., 2002)
	R: TCGGTCTGTAAATAAAGGTA			
*ebpS*	F: CAATCGATAGACACAAATTC	526	50	(Jarraud et al., 2002)
	R: CAGTTACATCATCATGTTTA			
*sea*	F: GAAAAAAGTCTGAATTGCAGGGAACA	560	58	(Peacock et al., 2002)
	R: CAAATAAATCGTAATTAACCGAAGGTTC			
*seb*	F: ATTCTATTAAGGACACTAAGTTAGGGA	404	57	(Peacock et al., 2002)
	R: ATCCCGTTTCATAAGGCGAGT			
*sec*	F: GTAAAGTTACAGGTGGCAAAACTTG	297	58	(Peacock et al., 2002)
	R: CATATCATACCAAAAAGTATTGCCGT			
*sed*	F: GAATTAAGTAGTACCGCGCTAAATAATATG	492	55	(Peacock et al., 2002)
	R: GCTGTATTTTTCCTCCGAGAGT			
*see*	F: CAAAGAAATGCTTTAAGCAATCTTAGGC	482	57	(Peacock et al., 2002)
	R: CACCTTACCGCCAAAGCTG			
*seg*	F: AATTATGTGAATGCTCAACCCGATC	642	60	(Jarraud et al., 2002)
	R: AAACTTATATGGAACAAAAGGTACTAGTTC			
*seh*	F: CAATCACATCATATGCGAAAGCAG	376	60	(Jarraud et al., 2002)
	R: CATCTACCCAAACATTAGCACC			
*sei*	F: CTCAAGGTGATATTGGTGTAGG	576	60	(Jarraud et al., 2002)
	R: AAAAAACTTACAGGCAGTCCATCTC			
*sej*	F: TAACCTCAGACATATATACTTCTTTAACG	300	60	(Jarraud et al., 2002)
	R: AGTATCATAAAGTTGATTGTTTTCATGCAG			
*sen*	F: ATGAGATTGTTCTACATAGCTGCAAT	680	60	(Jarraud et al., 2002)
	R: AACTCTGCTCCCACTGAAC			
*seo*	F: AGTTTGTGTAAGAAGTCAAGTGTAGA	180	60	(Jarraud et al., 2002)
	R: ATCTTTAAATTCAGCAGATATTCCATCTAAC			
*sem*	F: CTATTAATCTTTGGGTTAATGGAGAAC	300	60	(Jarraud et al., 2002)
	R: TTCAGTTTCGACAGTTTTGTTGTCAT			
*tst*	F: TTCACTATTTGTAAAAGTGTCAGACCCACT	180	51	(Peacock et al., 2002)
	R: TACTAATGAATTTTTTTATCGTAAGCCCTT			
*eta*	F: ACTGTAGGAGCTAGTGCATTTGT	190	57	(Peacock et al., 2002)
	R: TGGATACTTTTGTCTATCTTTTTCATCAAC			
*etb*	F: CAGATAAAGAGCTTTATACACACATTAC	612	54	(Peacock et al., 2002)
	R: AGTGAACTTATCTTTCTATTGAAAAACACTC			

### 2.6 Statistical analyses

The independent-samples method and a non-parametric test were used to estimate the correlation between *S. aureus* typing and various factors, including antimicrobial resistance, resistance genes, and virulence genes. Statistical analyses were performed using IBM SPSS Statistics (IBM Corp., Armonk, NY, USA) (*p* < 0.05 was considered to indicate statistical significance).

## 3 Results

### 3.1 Isolation and identification of *S. aureus*

**Figure 1:**
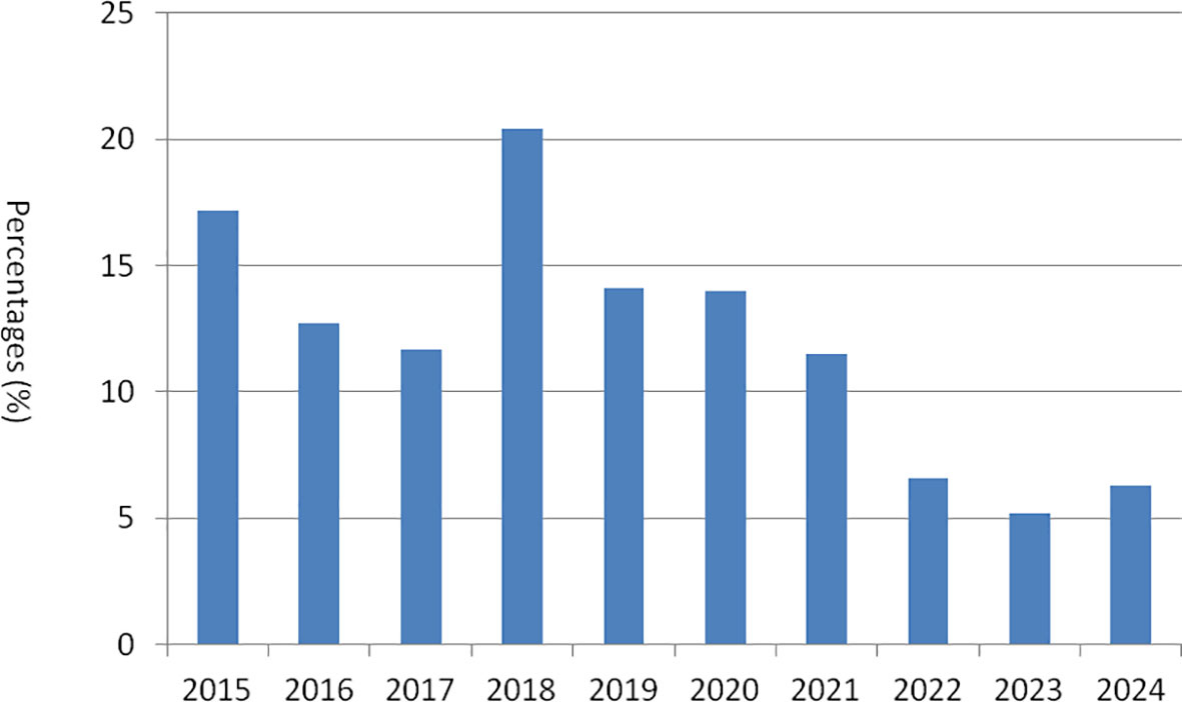
Prevalence of *S. aureus* strains from 2015 to 2024 in China.

In the present study, a total of 61 bacterial isolates were recovered in 515 milk samples and identified as *S. aureus* through Gram staining and species-specific PCR targeting the *nuc* gene. The *nuc* gene was successfully detected in all isolates. Upon sequencing, the homology of *nuc* gene sequences for five isolates in this study ranged from 98.6% to 100%, while the homology with other *S. aureus* strains available in GenBank ranged from 97.6% to 99.3%. The overall isolation rate was determined to be 11.8%. As shown in **Figure 1**, the highest prevalence rate was recorded in 2018, at 20.4% (11/54), followed by 17.2% (5/29) in 2015, and the lowest prevalence rate was observed in 2023, at 5.2% (3/58). Across five regions in China, the prevalence of the *S. aureus* infection rates was as follows (in descending order): 17.5% (10/57) in southern China, 13.6% (6/44) in eastern China, 12.0% (26/217) in central China, 11.0% (11/100) in northwestern China, and 8.2% (8/97) in northeastern China. Among the 14 provinces studied, Yunan province in southern China exhibited the highest isolation rate at 27.3%, while Gansu province in northwestern China had the lowest isolation rate at 6.3%. The details of the *S. aureus* isolates are documented in **Table 1**. The milking disinfection practices are shown in **Supplementary Table 1**. The cleaning agents, their concentrations, mode of application, time duration for each disinfection, and frequency of cleaning varied across different farms. Notably, some farms employing identical disinfection practices showed similar isolation rates (farms 3, 7, 8, 14, 20, 27, and 32), while others with the same disinfection practices demonstrated different isolation rates (farms 1, 2, 9, 11, 12, 16, 19, 21, 22, 26, and 28). Three farms (5, 10, and 17) utilizing low concentrations of disinfectants showed high isolation rates compared to those in farms 1, 9, 11, 21, and 28 that employed higher concentrations; however, these rates were comparable to those observed in three other farms (2, 12, and 19) using higher concentrations. Moreover, three farms (12, 22, and 31) that implemented a lower frequency of cleaning (twice per day) exhibited higher isolation rates than those in farms 1, 9, 11, 21, and 28 that maintained a higher frequency of cleaning (four times per day).

### 3.2 Bacterial typing

**Figure 2:**
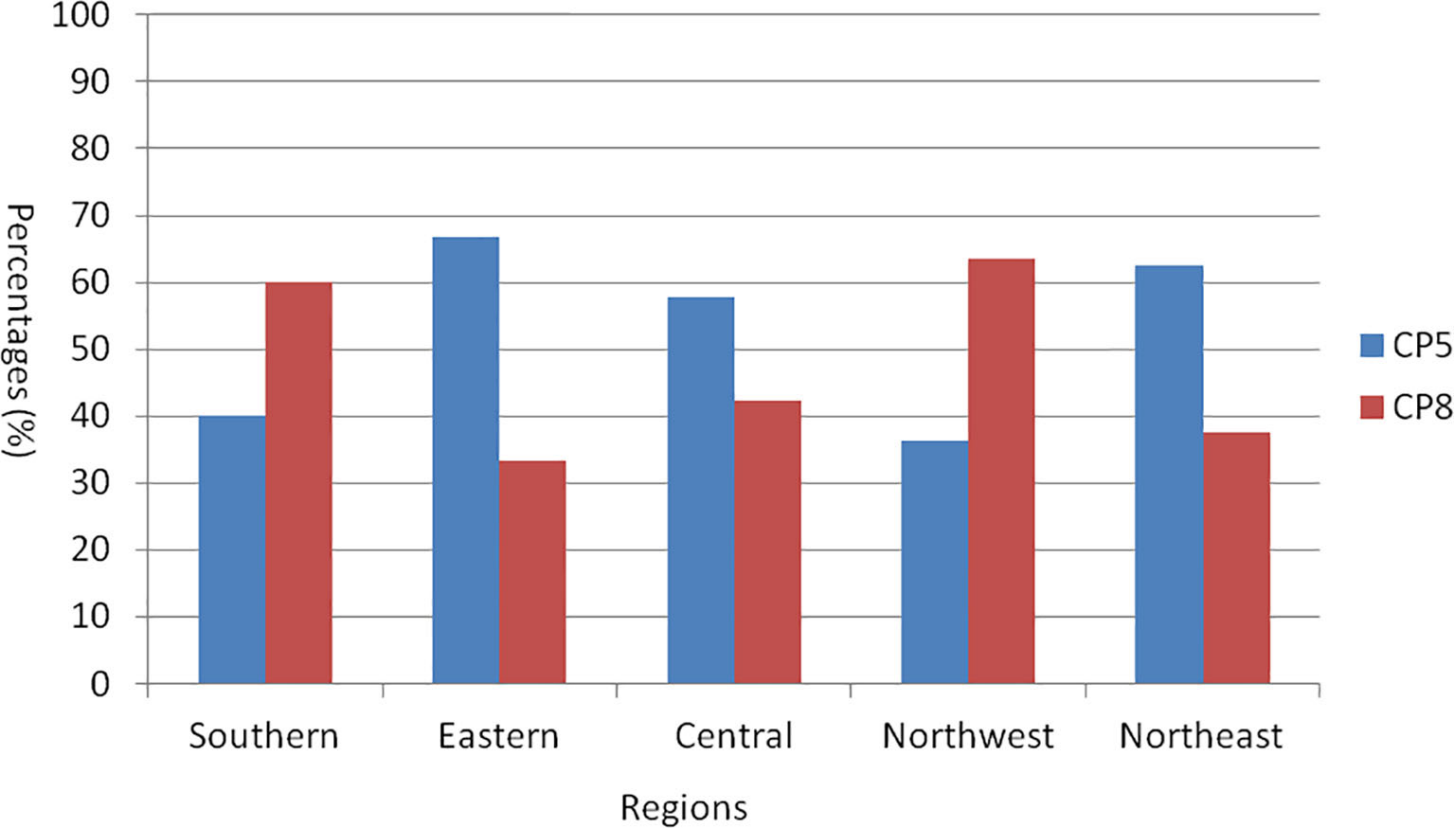
Capsular polysaccharide typing of 61 *S. aureus* strains present in five regions in China.

A total of 61 *S. aureus* isolates were classified into CP5 or CP8 types through PCR detection. The detection rates for CP5 in samples collected from various regions of China were as follows: southern China, 40.0% (4/10); eastern China, 66.7% (4/6); central China, 57.7% (15/26); northwestern China, 36.4% (4/11); and northeastern China, 62.5% (5/8). In contrast, the detection rates for CP8 in the same regions were 60.0% (6/10) in southern China, 33.3% (2/6) in eastern China, 42.3% (11/26) in central China, 63.6% (7/11) in northwest China, and 37.5% (3/8) in northeastern China (**Figure 2**).

**Figure 3:**
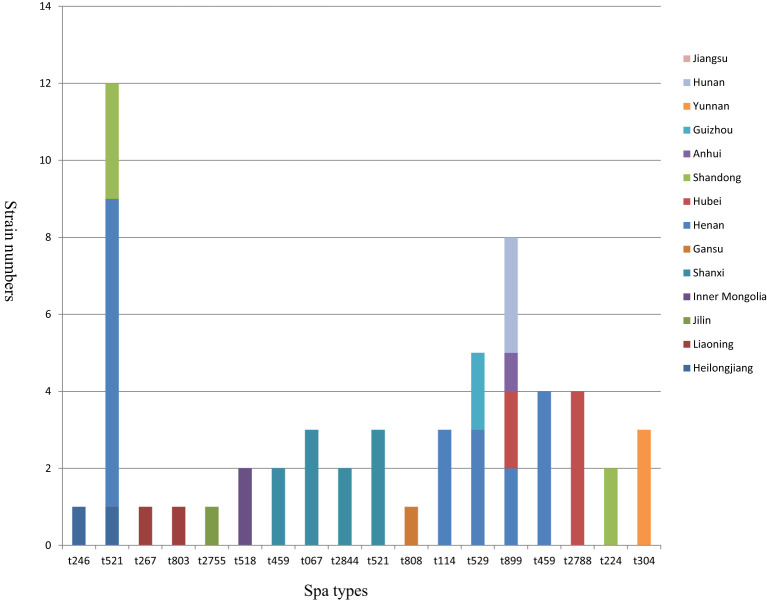
Distribution of 61 *S. aureus* strains typed by spa. Each column represents different spa types. The height of each column indicates the isolate number of the particular type. Each color represents a strain isolation site.

The various spa types and their corresponding geographical distributions are illustrated in **Figure 3**. A total of 61 *S. aureus* isolates were differentiated into 18 distinct spa types, with no novel spa types identified in this work. Among the 18 spa types, t521 was the most prevalent, accounting for 12 out of 61 isolates (19.7%), followed by t899 (8/61, 13.1%), t529 (5/61, 8.2%), t172 (4/61, 6.6%), t2788 (4/61, 6.6%), t067 (3/61, 4.9%), t870 (3/61, 4.9%), t114 (3/61, 4.9%), t304 (3/61, 4.9%), t518 (2/61, 3.3%), t459 (2/61, 3.3%), t2844 (2/61, 3.3%), t224 (2/61, 3.3%), t246 (1/61, 1.6%), t267 (1/61, 1.6%), t803 (1/61, 1.6%), t2755 (1/61, 1.6%), and t808 (1/61, 1.6%). In particular, the spa types t521 and t899 were the most widely distributed among goats, being detected in at least three provinces in China, whereas the other spa types exhibited more localized distributions.

### 3.3 Antimicrobial susceptibility

The 61 *S. aureus* strains isolated from dairy goats in this work were categorized as susceptible, intermediate, or resistant to 18 antibiotics belonging to eight classes. As shown in **Table 4**, isolates from different provinces exhibited varying degrees of resistance to the antibiotics tested. The resistance rates, ranked from highest to lowest, were as follows: penicillin (95.1%), enrofloxacin (82.0%), kanamycin (78.7%), levofloxacin (77.0%), oxacillin (75.4%), amikacin (73.8%), gentamicin (68.9%), tetracycline (62.3%), lincomycin (59.0%), erythromycin (47.5%), clindamycin (41.0%), doxycycline (39.3%), cefalotin (37.7%), ceftiofur (36.1%), chloramphenicol (29.5%), azithromycin (26.2%), sulfisoxazole (24.6%), and trimethoprim-sulfamethoxazole (11.5%). Additionally, 52 isolates (85.2%) were found to be multiresistant to three or more antibiotics, while one isolate (1.6%) demonstrated resistance to all antibiotics tested.

**Table 4:** The antimicrobial discs, breakpoints, and the distributions of antimicrobial resistances for 61 *S. aureus* strains isolated from goat milk samples with clinical mastitis.

Antimicrobial classes	Antimicrobial agent	Concentration (μg/piece)	The diameter of the bacteriostatic sphere			No. of isolates		
			Susceptible	Intermediate	Resistant	Susceptible	Intermediate	Resistant
β-lactams	Penicillin	10U	≥29	__	≤28	2 (3.28)	1 (1.6)	58 (95.1)
	Oxacillin	1	≥22	__	≤21	11 (18.0)	4 (6.6)	46 (75.4)
	Ceftiofur	30	≥24	__	≤20	29 (47.5	10 (16.4)	22 (36.1)
	Cefalotin	30	≥24	__	≤20	31 (50.8)	7 (11.5)	23 (37.7)
Sulfonamides	Sulfisoxazole	300	≥17	__	≤12	44 (72.1)	2 (3.28)	15 (24.6)
	Trimethoprim-sulfamethoxazole	1.25/23.75	≥16	__	≤10	54 (88.5)	0 (0)	7 (11.5)
Tetracyclines	Tetracycline	30	≥19	__	≤14	20 (32.8)	3 (4.9)	38 (62.3)
	Doxycycline	30	≥16	__	≤12	36 (59.0)	1 (1.6)	24 (39.3)
Macrolides	Erythromycin	15	≥23	__	≤13	28 (45.9)	4 (6.6)	29 (47.5)
	Azithromycin	15	≥18	__	≤13	42 (68.9)	3 (4.9)	16 (26.2)
Phenicols	Chloramphenicol	30	≥18	__	≤12	38 (62.3)	5 (8.2)	18 (29.5)
Aminoglycosides	Gentamicin	10	≥15	__	≤12	18 (29.5)	1 (1.6)	42 (68.9)
	Amikacin	30	≥17	__	≤14	16 (26.2)	0 (0)	45 (73.8)
	Kanamycin	30	≥18	__	≤13	13 (21.3)	0 (0)	48 (78.7)
Lincosamides	Lincomycin	2	≥21	__	≤14	21 (34.4)	4 (6.6)	36 (59.0)
	Clindamycin	2	≥21	__	≤14	34 (55.7)	2 (3.28)	25 (41.0)
Quinolones	Levofloxacin	5	≥19	__	≤15	13 (21.3)	1 (1.6)	47 (77.0)
	Enrofloxacin	5	≥19	__	≤15	11 (18.0)	0 (0)	50 (82.0)

The zone diameter (mm) interpretative criteria for drugs were interpreted in accordance with CLSI guidelines, and when antibiotics tested and interpreted are not available, the instructions for antibiotic-sensitive papers (Hangzhou Microbial Reagent Company, China) were used as reference.

In a further analysis from a geographical perspective, the average percentage of resistant strains to eight classes of antibiotics in five regions of China is recorded in **Table 5**. The results showed that isolates from all regions exhibited a higher sensitivity to sulfonamides, macrolides, and phenicols, while also being more resistant to quinolones and aminoglycosides. Notably, the average percentages were reobserved in the other four regions, with the overall average percentage of resistant strains across all antibiotic classes being the lowest at 46.1% (**Table 5**). In addition, in both CP5 and CP8 types, no significant difference was observed between *S. aureus* types and the antimicrobial resistance phenotypes among eight antimicrobial classes (*p* > 0.05).

**Table 5:** Average rates of antimicrobial resistance of *S. aureus* isolated from goat milk samples in five regions of China.

Antimicrobial classes	Average rates of antimicrobial resistance (%)				
	Northeast	Northwest	Central	East	South
β-lactams	59.4	61.4	66.3	54.2	52.5
Sulfonamides	18.8	18.2	15.4	16.7	25.0
Tetracyclines	50.0	59.1	53.8	41.7	45.0
Macrolides	37.5	36.3	40.4	41.7	25.0
Phenicols	37.5	27.3	30.8	33.3	20.0
Aminoglycosides	75.0	87.9	75.6	61.1	60.0
Lincosamides	43.8	50.0	53.8	58.3	40.0
Quinolones	75.0	72.7	88.5	66.7	75.0
Average	52.7	56.1	57.0	49.1	46.1

### 3.4 Detection of antimicrobial resistance genes

**Figure 4:**
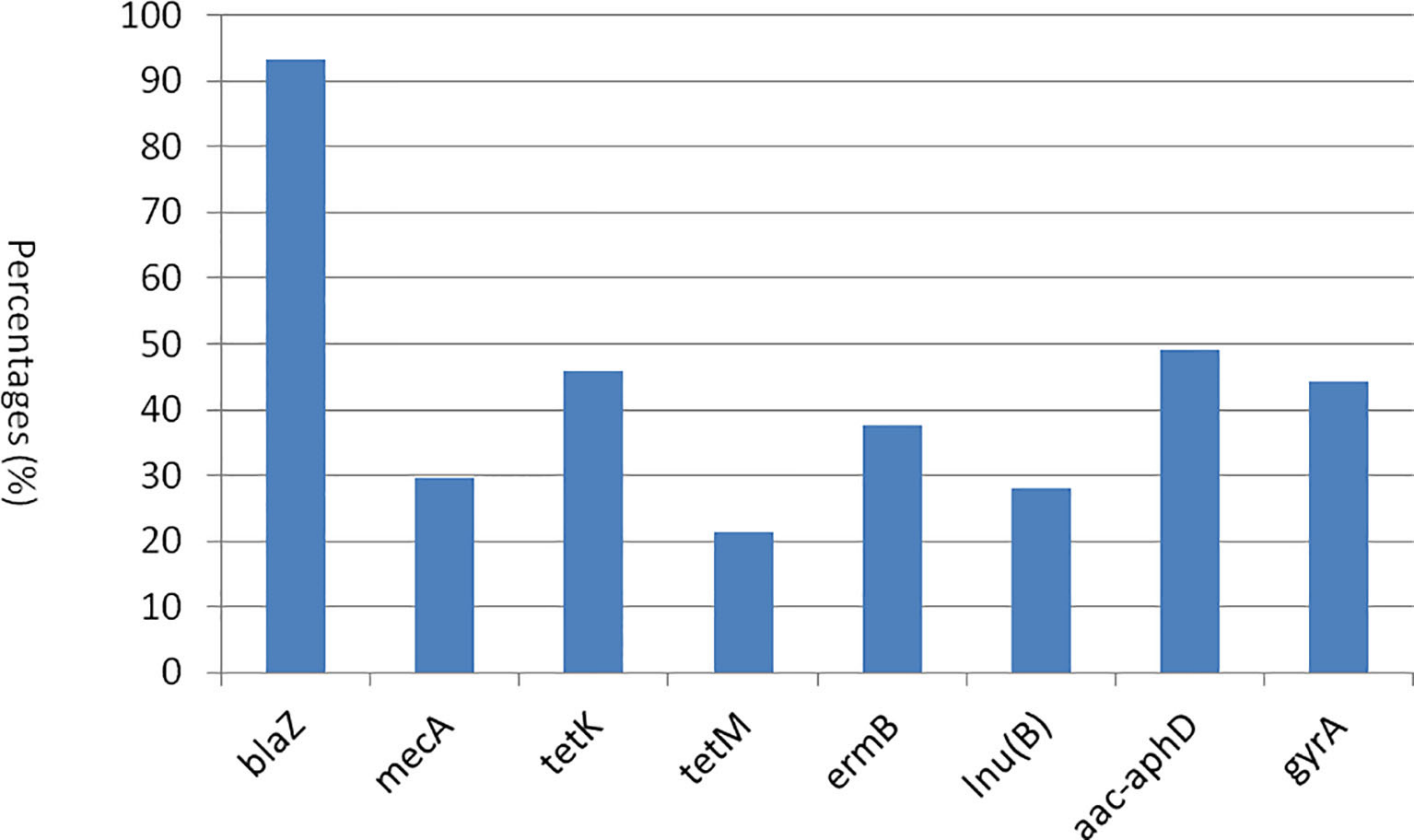
The distribution of antibiotic resistance genes among 61 *S. aureus* strains.

A total of 17 genes associated with resistance to eight distinct classes of antimicrobials were screened using PCR. Subsequently, eight genes from various classes, as well as multiple genes exhibiting a spectrum of resistance, were identified. Among the β-lactam resistance genes, *blaZ* was the most prevalent in 93.4% of the strains, followed by *mecA* at 29.5%. Notably, the *femA* and *femB* genes and *dfrD* genes were absent in the strains examined. In the context of tetracycline resistance, the *tetK* gene was more frequently observed (45.9%) compared to *tetM* gene (21.3%). Among the macrolide resistance genes, only the *ermB* gene (37.7%) was identified, with no detection of *ermA* and *ermC* genes. The *fexA* gene, which is associated with phenicol resistance, was absent in all strains analyzed. Furthermore, the *lnu(B)* gene, linked to lincosamide resistance, was found in 27.9% of the strains. The *aac-aphD* gene, associated with aminoglycoside resistance, was present in 49.2% of the samples. Moreover, among the quinolone resistance genes, only *gyrA* was detected (44.3%), while the *parC* gene was not found in any strains (**Figure 4**). As shown in **Supplementary Table 2**, 12 strains harbored only one resistance gene, whereas the remaining 49 strains possessed two or more resistance genes. Among the various patterns of multiple gene resistance, the combination of *blaZ* and *tetK* genes was the most prevalent, occurring in 6.6% of the strains. This was followed by the pattern comprising *blaZ*+*mecA*+*tetK*+*lnu(B*)+*aac-aphD*+*gyrA*, which was observed in 4.9% of the strains (3/61). Each of the seven additional patterns [*blaZ*+*mecA*+*tetM*, *blaZ*+*tetK*+*lnu(B*)+*aac-aphD*+*gyrA*, *blaZ*+*tetK*+*ermB*, *blaZ*+*mecA*+*ermB*+*aac-aphD*+*gyrA*, *blaZ*+*mecA*+*tetK*, *blaZ*+*tetK*+*ermB*+*aac-aphD*+*gyrA*, and *blaZ*+*tetK*+*lnu(B*)] was identified in two strains (3.3%). Most importantly, the remaining 28 strains exhibited unique patterns, each containing two or more antibiotic resistance genes. In this study, the detection of *blaZ*, *mecA*, *tetK*, *tetM*, *ermB, lnu(B*), *aac-aphD*, and *gyrA* in the corresponding antimicrobial resistance phenotypes was statistically significant (*p* < 0.05) (**Table 6**). Furthermore, in both CP5 and CP8 types, no significant difference was observed between *S. aureus* types and the antimicrobial resistance gene distributions among eight resistance genes (*p* > 0.05).

**Table 6:** Comparison of genotypic and phenotypic characteristics for antimicrobial resistance in *S. aureu*s isolates.

Antibiotics	Genes	Characteristics of *S. aureus* isolates		Association *p*
		G+ No(%)	P+ No(%)	
β-lactams	*blaZ*	57 (93.4%)	58 (95.1%)	0.008
	*mecA*	18 (29.5%)		0.254
Tetracycline	*tetK*	28 (45.9%)	42 (68.9%)	<0.001
	*tetM*	13 (21.3%)		0.005
Macrolide	*ermB*	23 (37.7%)	33 (54.1%)	<0.001
Lincosamide	*lnu(B)*	17 (27.9%)	41 (67.2%)	0.001
Aminoglycoside	*aac-aphD*	30 (49.2%)	49 (80.3%)	<0.001
Quinolone	*gyrA*	27 (44.3%)	52 (85.2%)	0.004

G+, gene positive; P+, phenotype resistance to antibiotics positive.

### 3.5 Detection of virulence genes

**Figure 5:**
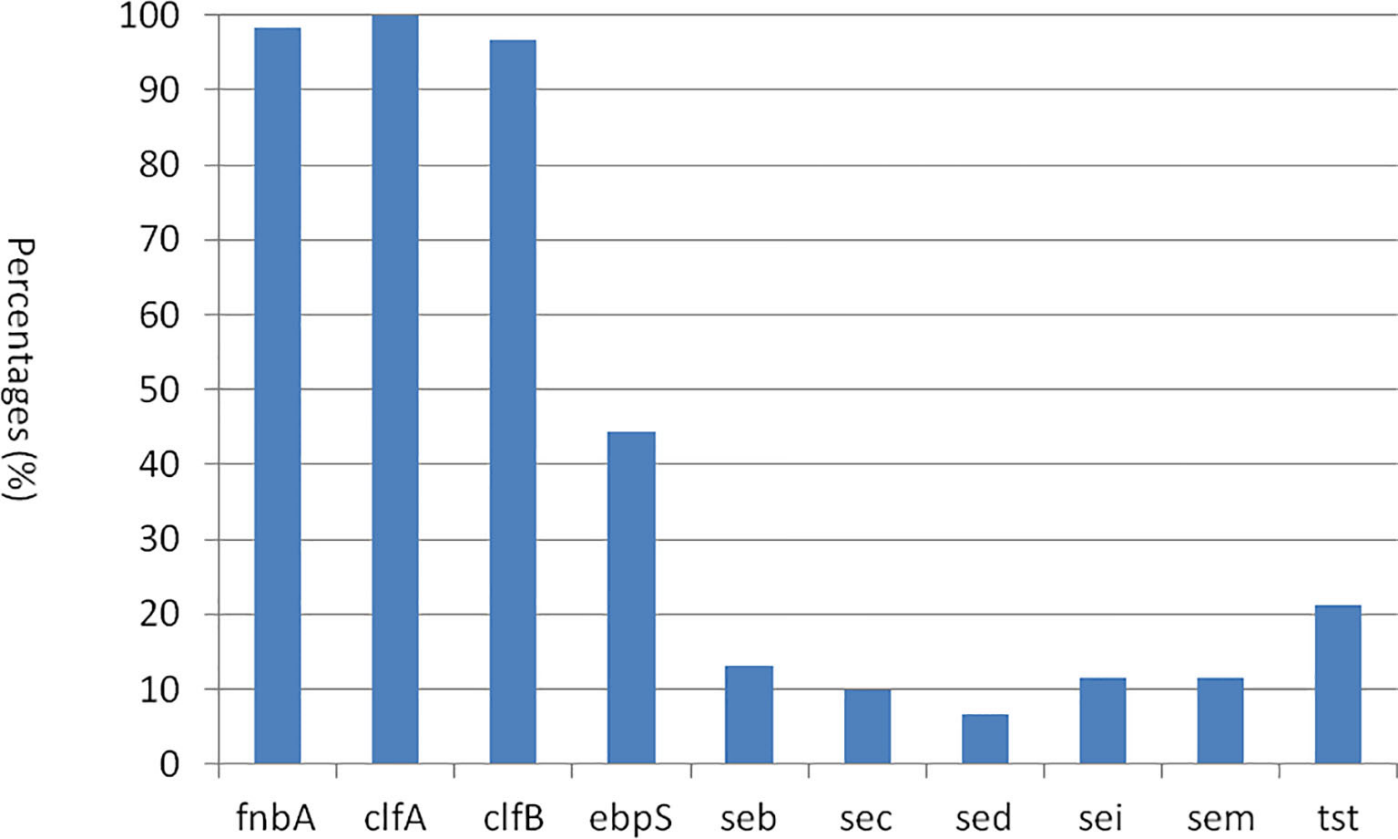
The distribution of virulence genes among 61 *S. aureus* strains.

The presence and distribution of virulence genes in 61 *S. aureus* strains are presented in **Figure 5**. All *S. aureus* isolates were found to possess the adhesin factor gene *clfA*, with a prevalence of 100% (61 strains). The majority of these strains also contained the *fnbA* gene (98.4%, *n* = 60) and *clfB* gene (96.7%, *n* = 59). In contrast, the *ebpS* gene was detected only in 44.3% (*n* = 27) of the strains. Regarding enterotoxin genes, the frequencies of *seb*, *sec*, *sed*, *sei*, and *sem* genes were observed in 13.1% (*n* = 8), 9.8% (*n* = 6), 6.6% (*n* = 4), 11.5% (*n* = 7), and 15.7% (*n* = 27) of the strains, respectively. Additionally, the toxic shock syndrome toxin gene *tst* was identified in 21.3% (*n* = 13) of the strains. Notably, none of the *S. aureus* isolates were positive for *sea*, *see*, *seg*, *seh*, *sej*, *sen*, *seo*, *eta*, and *etb* genes. In this study, in both CP5 and CP8 types, no significant difference was observed between *S. aureus* types and the virulence gene distributions among 10 positive virulence genes (*p* > 0.05).

## 4 Discussion

In this work, the nucleotide sequences of the nuc gene from the *S. aureus* isolates exhibited homology with other *S. aureus* strains in GenBank, ranging from 97.6% to 99.3%. Similar homology ranges were also observed in isolates from cows in China (Ning et al., 2023). The isolation rate of *S. aureus* was 11.8%, which is lower than the rates reported in goats from Shaanxi province (58.3%, 28/48) (Liu et al., 2023) and Yunnan province (15.24%, 32/209) (Zhao et al., 2015) in China, as well as lower than that found in goats in Indonesia (25.58%, 66/258) (Praja et al., 2023) and in Kenya (22.0%, 40/182) (Kabui et al., 2024). However, this rate is higher than that found in dairy goats in Portugal (6.9%, 35/508) (Andrade et al., 2021). Moreover, the isolation rate is comparable to those reported in four studies involving cows with mastitis in China, which reported rates of 10.1%, 11.1%, 10.1%, and 10.2%, respectively (Gao et al., 2017; Zhang et al., 2018; Zhang et al., 2022; Ning et al., 2023), and lower than the rate at 13.6% in Ningxia in China (Wang et al., 2017). The findings indicate that the prevalence of *S. aureus* in northeast and northwest China is lower than that observed in the other three regions and the overall average prevalence rate. Several management practices have been proven to be effective in controlling *S. aureus* mastitis, including post-milking teat dipping in antiseptic solutions and maintaining cows in a clean, dry, and suitable environment for the animals (Kerro Dego and Vidlund, 2024). These factors, as well as the use of antiseptic solutions, may influence the spread of *S. aureus*. Notably, isolation rates were low in most farms utilizing high concentrations of povidone iodine or sodium hypochlorite with extended disinfection times, with only a few exceptions exhibiting the opposite trend. These results suggest the necessity of selecting appropriate milking disinfection practices. Furthermore, other factors such as environmental temperature and humidity, the hygiene status of farms, and supplementation with vitamin E and selenium in goats (Ruegg, 2017) were not included in the analysis in this work. It is noteworthy that the prevalence of the pathogen has decreased to below 10% since 2022, likely due to the implementation of a national antimicrobial resistance monitoring and surveillance program. However, the limited number of samples in this study restricts the effectiveness of the evaluation, indicating that future investigations should include a larger sample size.

Capsular polysaccharide serotyping of *S. aureus* has been employed to predict and analyze the epidemiologic trends of isolates in dairy animals suffering from mastitis, with 11 serotypes documented in the literature (Poutrel et al., 1988). The detection rate of CP5 in our study was higher than previously reported in cattle in China, which were 42.1% (32/76) (Ning et al., 2023) and 35.57% (106/298) (Zhang et al., 2022), and also exceeded the rates observed in cattle in South Korea (Han et al., 2000). This information suggests that the distribution of CPs in *S. aureus* may be influenced by host species and geographical location. Most importantly, capsular polysaccharides are critical for the immunogenicity of *S. aureus* and have been proven to protect vaccinated mice from lethal challenges with the bacterium (Han et al., 2000). Our study provides a valuable reference for the development of vaccines against goat mastitis. Furthermore, among the 18 spa types in this study, t521 was the most prevalent, accounting for 19.7% (12/61) of the strains. This spa type has been previously detected in cattle at a rate of 2.01% in China, where it is considered a minor prevalent spa type (Zhang et al., 2022). Other notable spa types included t899 (13.1%, 8/61) and t529 (8.2%, 5/61), among others. These spa types have also been recorded in other reports involving isolates from cattle, at low detection rates (Zhang et al., 2022; Ning et al., 2023; Zhu et al., 2024). Additionally, isolates from cattle showed a high diversity of spa types. A national survey across 16 provinces identified 48 spa types among 298 *S. aureus* strains, with t459 as the predominant type (18.79%) (Zhang et al., 2022). In Hunan province, 20 spa types were identified among 76 *S. aureus* strains, with t796 as the most common type (23.68%) (Ning et al., 2023). Another investigation across 15 provinces found 17 spa types among 72 *S. aureus* strains, with t2734 as the main type (17.09%) (Zhu et al., 2024). Additionally, an investigation across nine provinces found 24 spa types among 103 *S. aureus* strains, with t359 as the main type (26.21%) (Zhang et al., 2018). However, among these three predominant types in goats, only t459 was detected at a rate of 3.3% in our study. In Greece, nine spa types were detected in 29 *S. aureus* strains, including t3586 (8), t4038 (6), t1534 (1), t306 (2), and t1773 (1) (Kotzamanidis et al., 2021); these types were not observed in the present study. Comparing these results, we speculate that *S. aureus* may spread between cattle and goat species, and isolates from different countries or regions often exhibit distinct molecular types. Notably, the dairy goats in this work were raised in backyard farms in a free-range style, sharing the same grassland with cattle, which underscores the necessity of controlling transmission between these two dairy species.

In China, antimicrobial therapy is the primary strategy for controlling goat mastitis; however, recent reports indicate an increase in bacterial resistance to antimicrobial agents among *S. aureus* isolates (Zhang et al., 2022; Ning et al., 2023; Zhu et al., 2024). Antimicrobial susceptibility testing is crucial for guiding the selection of appropriate antimicrobial agents for treating infections. In this work, the highest resistance was noted against penicillin, aligning with findings from several studies on *S. aureus* isolates in dairy cows with mastitis in China (Zhang et al., 2018; Cheng et al., 2019; Zhang et al., 2022). Given that penicillin is widely used in mastitis treatment, the selective pressure exerted on goats will likely accelerate the emergence of drug resistance. In contrast, studies conducted in Greece reported relatively low resistance rates for *S. aureus* isolates from goats, sheep, and cows, with rates of 6.9% (2/29), 16.5% (16/97), and 27.8% (10/36), respectively (Kotzamanidis et al., 2021). In Portugal, Staphylococcal isolates from goats and sheep exhibited a non-susceptible rate to penicillin of 27.7% (38/137) (Andrade et al., 2021). Our study indicated that the prevalence of penicillin-resistant *S. aureus* in dairy goat farms in China is higher than that observed in other countries. Additionally, in five regions, the isolates demonstrated high resistance to quinolones (ranging from 66.7% to 88.5%) and aminoglycosides (60.0% to 87.9%), which exceeded the resistance levels reported for bovine *S. aureus* in China (Zhang et al., 2022; Ning et al., 2023) and caprine *S. aureus* in Indonesia (Praja et al., 2023). This trend is not unexpected, as quinolones and aminoglycosides are among the most commonly used antibiotics in dairy animals in China (Wang, 2014). Their extensive use likely contributes to the increased resistance in *S. aureus*. Furthermore, the resistance rates of the isolates to the remaining five antimicrobial classes were lower than those observed in the three aforementioned antimicrobial classes, with sulfonamides exhibiting the lowest resistance rate. These findings underscore the urgency for judicious use of antimicrobials in the treatment of goat *S. aureus* mastitis. Therefore, clinical use of β-lactams, quinolones, and aminoglycosides should be minimized, unless guided by sensitive tests. Based on our results, sulfonamides should be considered as a preferential treatment option for *S. aureus* mastitis.

The two primary mechanisms employed by *S. aureus* to develop β-lactam resistance involve the synthesis of penicillinase associated with the *blaZ* gene and the production of penicillin-binding protein 2a linked to the *mecA*, *femA*, and *femB* genes (Kobayashi et al., 1994; Vannuffel et al., 1998; Choi et al., 2003; Olsen et al., 2006). In this work, the prevalence rates of *blaZ* and *mecA* genes were higher than the rates observed in water buffalo in Guangdong province, China (19.5% and 16.8%, respectively) (Zhang et al., 2023). These findings are, however, compared to those reported in cows in China, where the rates were 92.9% and 24.5%, respectively (Zhang et al., 2022), higher than the rates documented in cows in Pakistan (55.3% and 17.0%, respectively) (Haq et al., 2024). In *S. aureus* isolates, tetracycline resistance is mediated by the activation of efflux pumps encoded by the *tetM* and *tetK* genes (Al-Ashmawy et al., 2016; Merz et al., 2016; Akkou et al., 2018). Resistance to aminoglycosides is conferred by genes encoding aminoglycoside-modifying enzymes, such as *aac-aphD* gene (Strommenger et al., 2003). Quinolone resistance is associated with genes encoding the type II topoisomerase enzymes, specifically DNA gyrase (*gyrA*) and topoisomerase IV (*parC*) (Schmitt-Van-de-Leemput and Zadoks, 2007). Lincosamide resistance is mediated by the *lnu(B)* gene, which encodes a lincosamide inactivating nucleotidyl transferase (Bozdogan et al., 1999). As in this study, these resistance genes have also been detected in isolates from cows and water buffalo in China with varying prevalence rates (Zhang et al., 2022; Zhang et al., 2023). In isolates from goats in Pakistan, *mecA*, *blaZ*, *tetK*, *aac-aphD*, and *gyrA* genes were identified at rates of 35.9%, 45.6%, 32.5%, 50.49%, and 0%, respectively (Javed et al., 2024). To date, the documentation of antibiotic resistance genes in *S. aureus* isolates from goats in China is limited. Our data indicate that all isolates examined carry at least one resistance gene. Commonly, the correlation between antibiotic resistance genes and phenotypes was not significant (Bissong and Ateba, 2020), as several factors may affect bacterial antibiotic resistance phenotypes, including environmental changes, strain activity, and frequent use of antimicrobial agents. In contrast, genotypes are determined only by internal gene factors. However, in this study, the detection of *blaZ*, *mecA*, *tetK*, *tetM*, *ermB, lnu(B*), *aac-aphD*, and *gyrA* genes in the corresponding antimicrobial resistance phenotypes was statistically significant (*p* < 0.05). Similar correlations have also been observed in *S. aureus* isolates from cows in Hunan province in China (Ning et al., 2023). Although several isolates exhibited resistance to sulfonamides and phenicols, the corresponding resistance genes were not detected. This discrepancy may be attributed to the screening of only one or a few resistance genes. Consequently, a whole-genome sequencing approach in future research would provide a more comprehensive and unbiased antimicrobial resistance profile of these isolates. The high prevalence of antibiotic resistance genes among the 61 *S. aureus* isolates in this work underscores the issue of antibiotic misuse in dairy goats. It is imperative to exercise caution in antibiotic use and to monitor antibiotic-resistant genes in *S. aureus* isolates on farms.

Capsular polysaccharides have been identified as key pathogenicity factors associated with *S. aureus* (Han et al., 2000). The results of CP typing achieved a 100% positive rate. This finding is consistent with observations of *S. aureus* isolates from cows with mastitis in China (Zhang et al., 2022) and is notably higher than the detection rates reported in Jordan (91.3%) (Gharaibeh and Abu-Qatouseh, 2022), Australia (70.1%), and India (60.8%) (Gogoi-Tiwari et al., 2015). The lower detection rates in these regions may be attributed to the persistence of *S. aureus* in chronically infected hosts (Ambroggio et al., 2018). Adhesins are regarded as the most critical virulence factors during the initial stages of *S. aureus* infection, facilitating the invasion of host cells (Bien et al., 2011). In the present study, *clfA*, *fnbA*, *clfB*, and *ebpS* genes have been observed, which were also detected in isolates from cows (Zhang et al., 2018; Zhang et al., 2022) and from water buffalo (Zhang et al., 2023) in China. Enterotoxins are extracellular, low-molecular-weight proteins that exhibit resistance to heat, cold, and proteolytic enzymes (Suzuki et al., 2020), and they can elicit emetic and superantigenic activities in both animals and humans (Liu et al., 2022). In our research, the prevalence of *seb*, *sec*, *sed*, *sei*, and *sem* genes has been verified. A study involving strains from cows reported the prevalence of the *sea*, *seb*, *sec*, and *sed* genes at 17.5%, 16.4%, 7.4%, and 1.7%, respectively (Zhang et al., 2022). In Ningxia province, China, the prevalence of *sei*, *sen*, and *seu* was 44.0% each, followed by *seo*, *tst*, and *etB* (28.0% each), *etA* (24.0%), *sem* and *sep* (16.0% each), *seb*, *sec*, *sed*, and *sek* (12.0% each), and *sea* and *seh* genes (8.0% each); the *seg*, *sej*, and *ser* genes were present in 4.0% of the isolates (Wang et al., 2017). Another investigation of strains from water buffalo in Guangdong province, China, reported prevalence rates of 3.5% for *sea*, 15.9% for *sec*, and 9.7% for *see* gene (Zhang et al., 2023). These findings suggest that the enterotoxin gene content of *S. aureus* varies across different hosts and geographical regions. Toxic shock syndrome toxin, a prototypical superantigen encoded by the *tsst* gene, is known to induce toxic shock syndrome (Zhu et al., 2023). The prevalence of the *tsst* gene in *S. aureus* isolates from cows and water buffalo in China was reported to be 23.5% and 25%, respectively (Zhang et al., 2022; Zhang et al., 2023), which is higher than the rate observed in this study. Additionally, negative results for the virulence genes *eta* and *etb* were also noted in bovine isolates in China (Zhang et al., 2022). Therefore, it is imperative to monitor the epidemiology of virulence genes to mitigate public health risks.

## 5 Conclusions

The presence of *S. aureus* in goats with mastitis have been confirmed in 14 provinces. Notably, most *S. aureus* isolates exhibited multidrug resistance with diverse resistance patterns, and the diversity of antimicrobial resistance profiles associated with corresponding resistance genes (*p* < 0.05) was reported for the first time in *S. aureus* from caprine mastitis. Sulfonamides should be considered preferentially for the treatment of *S. aureus* mastitis. Additionally, spa types in this study have also been recorded in other reports involving isolates from cattle in China, suggesting potential cross-species transmissions of *S. aureus* between cattle and goats. Moreover, the high occurrence of the virulence genes *clfA*, *fnbA*, and *clfB* genes tested in this study in *S. aureus* underscores their pathogenic potential in causing mastitis in the studied area. Overall, this work provides a primary source for the molecular epidemiology of *S. aureus* in dairy goat herds in China.

## Data Availability

The original contributions presented in the study are included in the article/**Supplementary Material**. Further inquiries can be directed to the corresponding authors.
