# Supplementary material for: Characteristic profiles of molecular types, antibiotic resistance, antibiotic resistance genes, and virulence genes of Staphylococcus aureus isolates from caprine mastitis in China
Source: Front Cell Infect Microbiol. 2025 Feb 18;15:1533844. doi: 10.3389/fcimb.2025.1533844 (PMC11876158; doi:10.3389/fcimb.2025.1533844)
Supplement: Supplementary file 1 [file Table1.docx]

**Supplementary table 1** The milking disinfection practices in 32 farms

| Farm | Cleaning agents | Concentrations | Mode of application ^a^ | Time duration for each disinfection | Frequency of cleaning ^b^ |
| --- | --- | --- | --- | --- | --- |
| 1 | povidone iodine | 1% | Pre/post | 30s | 4/d |
| 2 | povidone iodine | 1% | Pre/post | 30s | 4/d |
| 3 | [sodium hypochlorite](javascript:;) | 3% | Pre/post | 30s | 2/d |
| 4 | [sodium hypochlorite](javascript:;) | 3% | Pre/post | 30s | 2/d |
| 5 | povidone iodine | 0.5% | Pre/post | 30s | 4/d |
| 6 | [potassium permanganate](javascript:;) | 0.02% | Pre/post | 20s | 4/d |
| 7 | [sodium hypochlorite](javascript:;) | 4% | Pre/post | 30s | 4/d |
| 8 | [sodium hypochlorite](javascript:;) | 3% | Pre/post | 30s | 2/d |
| 9 | povidone iodine | 1% | Pre/post | 30s | 4/d |
| 10 | povidone iodine | 0.5% | Pre/post | 30s | 4/d |
| 11 | povidone iodine | 1% | Pre/post | 30s | 4/d |
| 12 | povidone iodine | 1% | Pre/post | 30s | 2/d |
| 13 | povidone iodine | 0.5% | Pre/post | 30s | 4/d |
| 14 | [sodium hypochlorite](javascript:;) | 3% | post | 30s | 1/d |
| 15 | [chlorhexidine](javascript:;) | 0.5% | post | 30s | <1/d |
| 16 | povidone iodine | 1% | Pre/post | 20s | 4/d |
| 17 | povidone iodine | 0.5% | Pre/post | 30s | 4/d |
| 18 | [sodium hypochlorite](javascript:;) | 3% | Pre/post | 30s | 2/d |
| 19 | povidone iodine | 1% | Pre/post | 20s | 4/d |
| 20 | [sodium hypochlorite](javascript:;) | 3% | Pre/post | 30s | 2/d |
| 21 | povidone iodine | 1% | Pre/post | 20s | 4/d |
| 22 | povidone iodine | 1% | post | 20s | 2/d |
| 23 | [chlorhexidine](javascript:;) | 0.5% | pre | 20s | 2/d |
| 24 | [chlorhexidine](javascript:;) | 0.5% | pre | 20s | 2/d |
| 25 | [sodium hypochlorite](javascript:;) | 3% | Pre/post | 30s | 2/d |
| 26 | povidone iodine | 1% | Pre/post | 20s | 4/d |
| 27 | [sodium hypochlorite](javascript:;) | 3% | Pre/post | 30s | 2/d |
| 28 | povidone iodine | 1% | Pre/post | 30s | 4/d |
| 29 | [potassium permanganate](javascript:;) | 0.02% | pre | 30s | 1/d |
| 30 | [sodium hypochlorite](javascript:;) | 3% | Pre/post | 30s | 4/d |
| 31 | povidone iodine | 0.5% | pre | 20s | 2/d |
| 32 | [sodium hypochlorite](javascript:;) | 3% | Pre/post | 30s | 2/d |

^a^: pre: pre-milking teat disinfection; post: post-milking teat disinfection; Pre/post: pre-milking teat disinfection and post-milking teat disinfection.

^b^: 1/d:once per day; <1/d: less than once per day; 2/d: twice per day; 4/d: four times per day.

**Supplementary table 2** Antimicrobial resistance genes distributions among 61 strains

| Strains/Antibiotic resistance genes | blaZ | mecA | tetK | tetM | ermB | lnu | aac |
| --- | --- | --- | --- | --- | --- | --- | --- |
| 1 | + ^a^ | - ^b^ | + | - | - | + | + |
| 2 | + | + | - | - | + | + | + |
| 3 | + | - | - | - | - | + | - |
| 4 | + | + | + |  | + | - | + |
| 5 | + | - | - | - | - | - | - |
| 6 | + | - | - | - | - | - | - |
| 7 | + | + | - | + | + | - | + |
| 8 | + | - | + | - | - | - | + |
| 9 | - | - | - | + | + | - | + |
| 10 | + | - | - | - | - | - | - |
| 11 | + | - | - | - | - | - | - |
| 12 | + | - | + | - | + | + | + |
| 13 | + | + | - | + | - | - | - |
| 14 | + | - | + | - | - | + | + |
| 15 | + | + | + | - | + | - | + |
| 16 | + | - | + | + | - | - | - |
| 17 | + | + | - | - | - | + | + |
| 18 | + | - | + | - | + | - | - |
| 19 | + | + | + | - | - | - | + |
| 20 | + | - | - | - | + | + | + |
| 21 | + | + | - | + | - | - | - |
| 22 | - | - | - | - | + | + | + |
| 23 | + | - | - | - | - | - | - |
| 24 | + | - | + | - | - | + | + |
| 25 | + | - | - | - | - | - | - |
| 26 | + | + | - | - | + | - | + |
| 27 | + | - | + | - | - | - | - |
| 28 | + | - | + | + | + | + | + |
| 29 | + | - | - | - | - | - | - |
| 30 | + | + | + | - | - | - | - |
| 31 | + | - | - | - | + |  | + |
| 32 | + | + | + | - | - | - | - |
| 33 | + | - | - | + | + | + | + |
| 34 | + | - | - | - | + | - | - |
| 35 | - | - | + | - | - | - | - |
| 36 | + | - | - | - | + | + | + |
| 37 | + | + | - | - | - | - | - |
| 38 | + | - | + | + | - | - | + |
| 39 | + | - | + | - | - | - | - |
| 40 | - | - | - | + | - | + | + |
| 41 | + | - | + | - | - | - | - |
| 42 | + | - | - | - | - | - | - |
| 43 | + | - | + | - | + | - | + |
| 44 | + | - | - | + | - | - | - |
| 45 | + | + | - | - | + | - | + |
| 46 | + | - | + | - | - | + | - |
| 47 | + | - | - | + | - | - | - |
| 48 | + | - | + | - | + | - | - |
| 49 | + | + | + | - | - | + | + |
| 50 | + | - | - | + | + | - | - |
| 51 | + | - | + | - | + | - | + |
| 52 | + | - |  | - | - | - | - |
| 53 | + | + | + | - | - | - | + |
| 54 | + | - | + | - | - | + | - |
| 55 | + | + | + | - | + | - | + |
| 56 | + | - | - | + | - | - | + |
| 57 | - | - | - | - | + | - | - |
| 58 | + | - | + | - | - | - | - |
| 59 | + | + | + | - | - | - | + |
| 60 | + | - | - | - | - | - | - |
| 61 | + | + | - | - | + | + | + |

^a^:+: positive for detected gene.

^b^: -: negative for detected gene.
